# Spin‐Noise‐Detected Two‐Dimensional Nuclear Magnetic Resonance at Triple Sensitivity

**DOI:** 10.1002/cphc.201800008

**Published:** 2018-02-20

**Authors:** Stephan J. Ginthör, Kousik Chandra, Matthias Bechmann, Victor V. Rodin, Norbert Müller

**Affiliations:** ^1^ Institute of Organic Chemistry Johannes Kepler University Linz Altenbergerstraße 69 4040 Linz Austria; ^2^ Present address: NMR Research Centre Indian Institute of Science Bangalore 560012 India; ^3^ Faculty of Science University of South Bohemia Branišovská 1645/31A 370 05 České Budějovice Czech Republic

**Keywords:** cross-correlation, fast acquisition, multiple-quantum coherence, nuclear spin noise, two-dimensional nuclear magnetic resonance

## Abstract

A major breakthrough in speed and sensitivity of 2 D spin‐noise‐detected NMR is achieved owing to a new acquisition and processing scheme called “double block usage” (DBU) that utilizes each recorded noise block in two independent cross‐correlations. The mixing, evolution, and acquisition periods are repeated head‐to‐tail without any recovery delays and well‐known building blocks of multidimensional NMR (constant‐time evolution and quadrature detection in the indirect dimension as well as pulsed field gradients) provide further enhancement and artifact suppression. Modified timing of the receiver electronics eliminates spurious random excitation. We achieve a threefold sensitivity increase over the original snHMQC (spin‐noise‐detected heteronuclear multiple quantum correlation) experiment (K. Chandra et al., *J. Phys. Chem. Lett*. **2013**, *4*, 3853) and demonstrate the feasibility of spin‐noise‐detected long‐range correlation.

Spin‐noise‐detected 2 D NMR was introduced as a proof‐of‐concept recently.^1^ It is based on the phenomenon of nuclear spin noise, which Felix Bloch predicted in 1946 as a consequence of the statistically incomplete cancellation of random fluctuations^2^ in finite sized spin ensembles. Its first experimental observation had to wait until 1985,^3^, ^4^ because of the low signal amplitudes and, compared to today's standards, less sophisticated spectrometer hardware. Recent advances in the field, especially the wide availability of cryogenically cooled probes,^5^ allowed the measurement of spin noise on many spectrometers and renewed the interest in this phenomenon.^6^, ^7^, ^8^ Even if practical applications in spectroscopy are still very limited, the properties of spin noise seem a promising approach for addressing certain types of problems. Extensive research in one‐dimensional spin noise spectroscopy gave rise to various novel application in the fields of spectroscopy and imaging.^7^ Spin noise is also relevant for interference‐free investigations of spin systems.^9^ The ultimate promise, however, is the realization of nano‐scale level (less than 10^8^ spins) NMR spectroscopy, in a range where noise magnetization dominates over thermal polarization‐based magnetization.^3^, ^10^ Our focus is set on spectroscopic applications for liquid samples.^1^, ^7^, ^8^, ^9^, ^11^, ^12^, ^13^ To date, spin‐noise‐detected NMR spectroscopy is still in an early stage of development and will require additional hardware advances for implementation at nano‐scale. However, in magnetic resonance force microscopy (MRFM) spin noise detection has become the state‐of‐the art.^14^ Issues of other noise sources affecting NMR spin noise spectra have been resolved recently^8^ and are briefly discussed in the Supporting Information.

The first reported 2 D spin‐noise‐detected spectrum was the snHMQC (spin‐noise‐detected heteronuclear multiple quantum correlation) experiment,^1^ which is based on the pairwise cross‐correlation of noise blocks that symmetrically sandwich the mixing and evolution periods of a heteronuclear 2 D experiment. The general timing diagram of a conventional radio frequency (RF) pulse‐excited 2 D NMR experiment is compared to the ones of spin‐noise‐detected 2 D NMR experiments in Figure 1.

**Figure 1:**
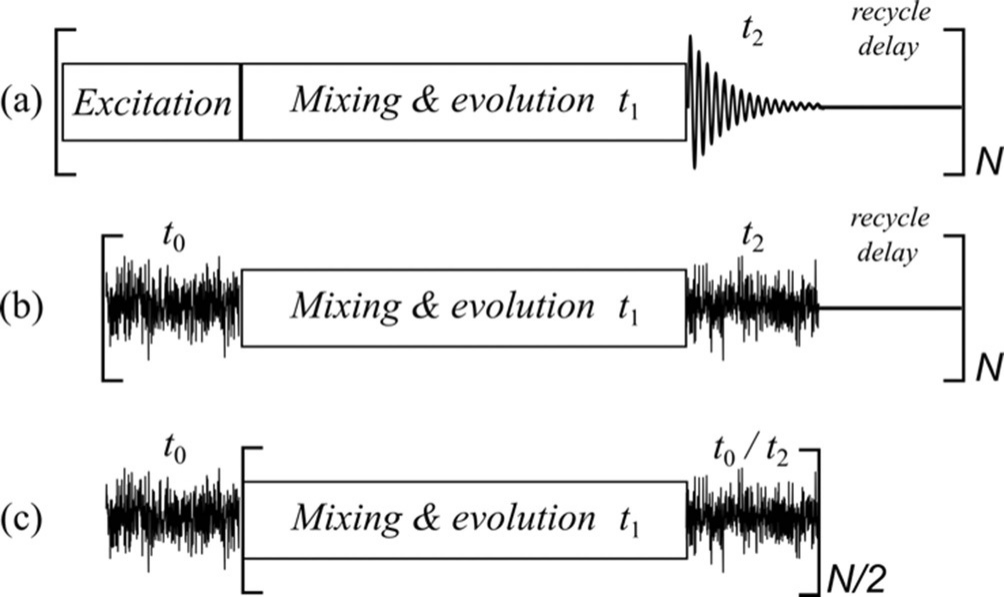
a) Generalized timing scheme of a pulse‐excited 2 D NMR experiment. *t*_2_ denotes the acquisition block recording the FID. The period *t*_1_ represents the indirectly detected dimension. Coherence transfer occurs during excitation and mixing periods. b) Acquisition scheme of a spin‐noise‐detected 2 D NMR experiment. *t*_0_ and *t*_2_ both denote acquisition blocks, where the so‐called noise blocks are recorded. Correlated together, they correspond to the directly detected dimension *t*_2_ of scheme (a). c) Efficient fast acquisition scheme for spin‐noise‐detected 2 D NMR experiments with DBU halves the number of noise block recordings. The mixing and evolution periods and the acquisition block labelled *t*_0_/*t*_2_ are repeated as required to obtain sufficient signal‐to‐noise ratio. Recycle delays are completely omitted with this scheme, which further reduces the total experimental time.

For a conventional 2 D NMR experiment, as drawn in Figure 1 a, the spin coherences are excited by one or more RF pulses, modulated during the evolution period, transferred in the mixing period(s) and detected during *t*_2_. During evolution the amplitudes or phases of the coherences are modulated with a characteristic frequency, which may be a chemical shift, coupling constant or a linear combination of these.^15^ In spin‐noise‐detected experiments, as schematically drawn in Figure 1 b, no RF irradiation is applied to the detected spins (^1^H in most cases), as both excitation and detection rely exclusively on intrinsic random spin fluctuations, which are usually called spin noise and are intrinsic to the tightly coupled system consisting of the nuclear spins and the resonance RF circuit.^8^ For each repetition of the 2 D pulse sequence, two input and output noise blocks are acquired and cross‐correlated as demanded by the nature of the stochastic excitation process. Cross‐correlation is achieved as detailed in the paper by Chandra et al.^1^ and illustrated in the table of contents graphics. The two noise blocks (*t*_0_ and *t*_2_) are Fourier transformed first and then the resulting spectra are combined by point‐wise complex‐conjugate multiplication, which owed to the Einstein–Wiener–Khinchin theorem,^16^ is equivalent to cross‐correlation followed by Fourier transformation. For a 2 D experiment this has to be done for each individual point in the indirect time domain *t*_1_ before summation of the repetitions. Fourier transform along *t*_1_ yields the final spin‐noise‐detected 2 D NMR spectrum, which can be phase sensitive in the indirect frequency dimension.

The first actual implementation of the general scheme, the snHMQC experiment^1^ is shown in Figure 2 a. Even though there are no RF pulses applied on the observed proton (^1^H) channel, they are still needed on the carbon (^13^C) channel.

**Figure 2:**
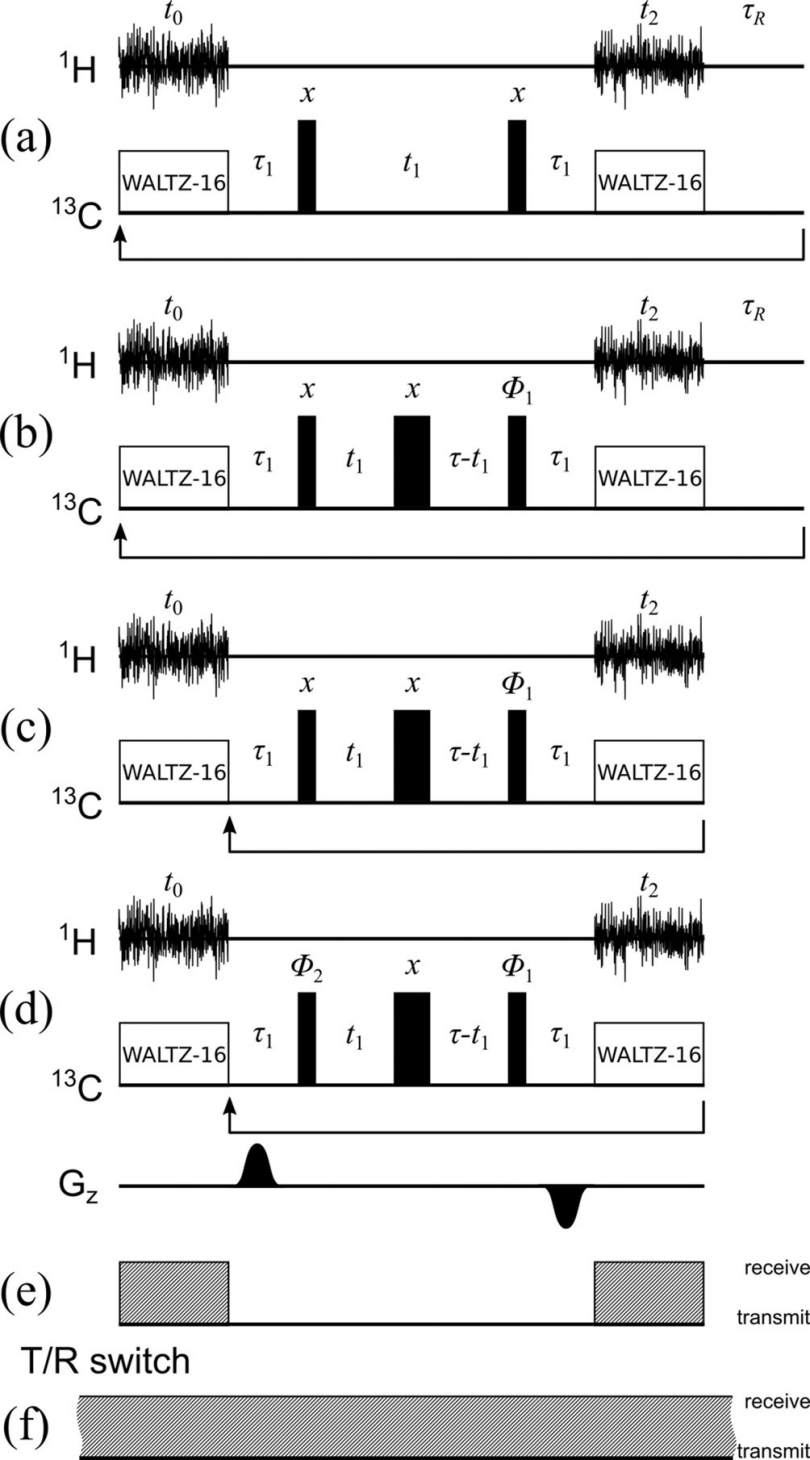
Pulse sequences for the original^1^ and enhanced snHMQC and snHMBC experiments. *t*_0_ and *t*_2_ denote the two acquisition blocks. *τ*_1_ is nominally set to 1/(2*J*_CH_) but can be optimized for relaxation and passive couplings (*J*_CH_ denotes the heteronuclear coupling constant). *t*_1_ is the evolution time (^13^C). WALTZ‐16 decoupling is indicated by the correspondingly labeled blocks. Narrow and wide black rectangles denote hard 90° and 180° RF pulses, respectively. The data collected during the acquisition blocks are stored separately. Panel (a) shows the original snHMQC experiment^1^. Panel (b) shows the pulse sequence for the constant‐time ctsnHMQC experiment. The constant time *τ* is nominally set to 1/*J*_CC_ (*J*_CC_ denotes the homonuclear carbon coupling constant) but can be optimized for relaxation and competing couplings as given in the Experimental Section. The phase *Φ*_1_ is 0° in the basic sequence. For States‐TPPI quadrature detection, first *N* noise blocks are recorded with *Φ*_1_=0°, then with *Φ*_1_=90°. Upon each incrementation of *t*_1_, *Φ_1_* is incremented by 180°. Panel (c) shows the ctsnHMQC experiment with DBU. Panel (d) shows the pulse sequence of the ctsnHMBC experiment. The changes from ctsnHMQC are the addition of *z*‐gradient pulses and the first 90° pulse also having a variable phase *Φ_2_*. Each setting of *Φ*_1_ is recorded with *Φ_2_*=0° and *Φ*_2_=180°, while *Φ*_1_ does not apply TPPI anymore (no phase increment per *t*_1_ increment). Panel (e) shows the classical way of switching the preamplifier from transmit to receive mode. Panel (f) shows the new scheme avoiding preamplifier mode switching in order to prevent transmit/receive (T/R) switching artifacts.

The tiny amplitudes of spin noise signals make it mandatory to repeat the accumulation very often (typically over 1000 times) for each *t*_1_ value. The separation of signals due to coherence transfer from spin noise during *t*_0_ on the one side, and de novo spin noise or random electronic noise in *t*_2_ on the other side, can be achieved because, even though the phases of all contributions are random, the components that undergo coherence transfer via the mixing and evolution periods have constant relative phases in *t*_0_ and *t*_2_. In order to improve the efficiency of spin‐noise‐detected multi‐dimensional NMR our goal was to improve the sensitivity and efficiency (signal‐to‐noise in a given time interval) of these experiments substantially.

Introducing constant‐time evolution (Figure 2 b) achieves several improvements. Firstly, constant‐time evolution avoids the splitting by homonuclear ^13^C–^13^C coupling in the indirect dimension, which is relevant when using uniformly ^13^C labeled samples. The choice of the constant‐time interval *τ* depends on all heteronuclear couplings and relaxation of multiple quantum coherence. It can be optimized to give a maximum in‐phase coherence at the end of the evolution period. In practice, the mixing and evolution periods should be kept as short as possible to minimize loss of residual coherence between the first noise block (*t*_0_) and the second one (*t*_2_). Secondly, constant‐time evolution provides for quadrature detection in the indirect dimension as outlined later. In the acquisition scheme of Figure 1  with actual pulse sequences shown in Figure 2 a and b, noise blocks *t*_0_ and *t*_2_ are recorded separately for each of the *N* repetitions, yielding *N* cross‐correlated spectra to accumulate for each *t*_1_ value. After each acquisition of a *t*_2_ noise block, there is a recycle delay *τ_R_*, followed by the acquisition of the *t*_0_ noise block of the next repetition. As, relying on spin noise, there is no need to wait for any spin system recovery, one can skip the recycle delay within spectrometer system's timing requirements. With data acquired in this way, during processing every noise block (except the first and last one) can be used as a *t_2_* block for one and as a *t_0_* block for the subsequent cross‐correlation computation.

This effectively means repeating the basic sequence (“noise acquisition–mixing–evolution–mixing”) without delay (Figure 1 c), a fast acquisition scheme we denote “double block usage” (DBU). Any magnetization coherently transferred between *t*_0_ and *t*_2_, that has not fully decayed by the end of one *t*_2_ acquisition block, will undergo another “mixing–evolution–mixing sequence” until it decays below any reasonable detection limit. Conversely, the de novo spin noise originating during the *t*_2_ period will cross‐correlate with the next noise block as before. With the original scheme (Figure 1 b), *N* recorded noise blocks yield *N/2* cross‐correlations. The DBU scheme increases this number to *N*−1. For larger *N* the number of cross‐correlations thus is practically increased by a factor of two. As the accumulated spin noise signal increases linearly with the number *N* of cross‐correlated spectra added, while the background noise only increases with the √*N*, this results in a factor √2 improvement of the signal‐to‐noise ratio over the original approach.^1^ Additionally, the elimination of the recycle delays reduces the total experiment time further.

We implemented DBU in the ctsnHMQC (constant‐time snHMQC) experiment also adding quadrature detection in the indirect dimension using the States‐TPPI (time‐proportional phase incrementation^17^, ^18^) approach, by varying the phase *Φ*_1_ accordingly, as shown in Figure 2 c. Thus signals, which are not modulated during *t*_1_ are moved to the edge of the spectrum. The phase sensitive detection enables one to combine parallel coherence transfer pathways to yield a single peak (as opposed to the double and zero quantum coherence cross peaks (*Ω*_H_±*Ω*_C_) of the original experiment) so that another √2 sensitivity improvement is achieved.

In the long‐range correlation experiment (ctsnHMBC, Figure 2 d) we use additional pulsed field gradients during the mixing periods, which is due to the following rational: During the *τ_1_* period the ^1^H transverse magnetization coupled to ^13^C evolves into antiphase coherence. The pulsed field gradient during this period spatially disperses transverse magnetization thus reducing the amount or radiation damping,^19^ which would otherwise quench the signal available for cross‐correlation. A gradient of opposite sign in the next *τ_1_* period refocuses only these coherences, while others, which would enhance radiation damping, are dispersed. The introduction of the pulsed field gradients thus improves the signal‐to‐noise ratio especially for long mixing times. Apart from counteracting radiation damping, the sign alternating gradient pulses defocus potential signals originating from noise originating between the detection periods. These would contribute to the uncorrelated noise in the spectrum and thus reduce its signal‐to‐noise ratio. It should be noted that, when using gradients with the fast DBU technique, the fast repetition of gradient may prevent the operation of the spectrometer's field‐frequency lock. To mitigate this interference, extra delays between incrementations of *t_1_* may be required to allow for magnetic field re‐optimization. Their duration is however negligible compared to the total experiment time. The states scheme for quadrature detection is used for the ctsnHMBC experiment, too. However, TPPI is replaced by a simple axial peak suppression scheme using 0°/180° alternating phase for *Φ*_2_ (on the first 90° pulse) and subtraction of the resulting spectra (after 1 D FT, correlation and summation) during processing so the correlated signals add up constructively. Thus, for each *t_1_* value, there are four sets of repetitions (instead of two used in ctsnHMQC) alternating *Φ*_1_ between 0° and 90° and *Φ*_2_ between 0° and 180° independently. It should be noted that in this type of experiment each phase cycling step has to be stored separately as is also the case in multiplex NMR methods.^20^

While testing spin‐noise‐detected experiments on different spectrometers we noticed evidence of spurious excitation on some hardware configurations. The experiments documenting that behavior are laid out in the Supporting Information. In summary, the active impedance switching of the preamplifier between a low impedance (50 Ω) “pulse mode” and a high impedance “receive mode” can cause minute signal excitation of random phase and amplitude occurring at the moment of activation of this transmit/receive (T/R) switch. The common T/R switching scheme implemented in the standard acquisition schemes can be seen in Figure 2 e. To avoid any influence of impedance switching all new experiments shown here were using new implementations of the pulse programs explicitly programming the T/R switch to remain in receive mode all the time, which is shown in Figure 2 f. The pulse programs are available in the Supporting Information.

In Figure 3 we compare the first spin‐noise‐detected 2 D NMR spectrum^1^ to the improved version (ctsnHMQC, Figure 2 c) with all improvements outlined above applied.

**Figure 3:**
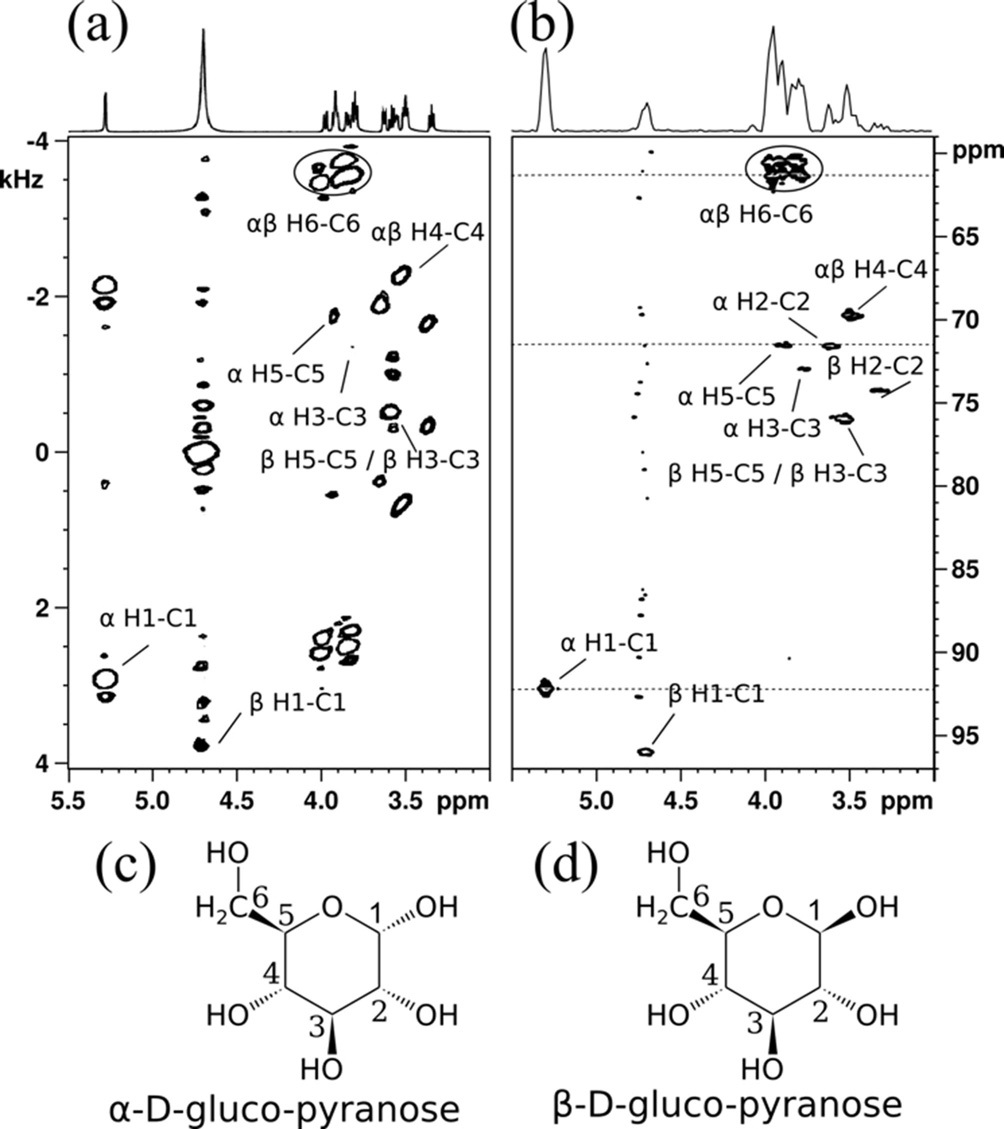
Panel (a) depicts the first 2 D spin‐noise‐detected (snHMQC) spectrum published by Chandra et al.^1^ (recording time: 40 h). The 1 D trace is a standard ^1^H spectrum. In panel (b), we show the result of the current implementation of the ctsnHMQC experiment according to Figure 2 c and e applying all enhancements laid out in the text. Total recording time was 20 h. Note the absence of the zero quantum (*Ω*_H_−*Ω*_C_) peaks in panel (b). The 1 D spectrum is a projection of the 2 D spectrum. Horizontal dashed lines mark the locations of the cross sections shown in Figure 4. The numbering of the prevailing isomers of glucose in aqueous solution is shown in panel (c) for the α‐d‐pyranose and in panel (d) for the corresponding β anomer.

Even though the total recording time was shrunk from the original 40 h to just 20 h, the signal‐to‐noise ratio could be improved by a factor of approximately √2. This is owed to the combined effects of the DBU and phase sensitive quadrature detection both contributing a factor of approximately √2. Additionally, the faster recycle time and lower requirement of long‐term stability as well as the elimination of spurious excitation background, the improvement exceeds the theoretical factor of 2 slightly. All single bond hydrogen‐carbon coupling pairs of both anomeric forms of the gluco‐pyranose could be identified in the new faster and more sensitive version of the experiment. It is also noteworthy, that due to the improvements of the recording technique, the *t*_1_ noise artifacts visible at 4.7 ppm (from residual HOD) in Figure 3 a are absent from the improved spectrum of Figure 3 b.

In Figure 4 we illustrate the gain in signal‐to‐noise ratio obtained through the DBU scheme by comparing three cross sections obtained from the same raw data as Figure 3 b. The traces on the left side were obtained without DBU. The spin noise signal power amplitudes in the right side traces are approximately doubled owed to the use of DBU. As the uncorrelated noise power also increases by a factor √2 the signal‐to‐noise ratio rises by √2, too.

**Figure 4:**
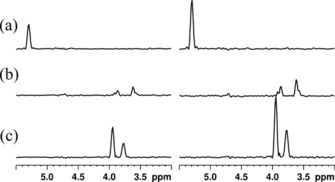
^1^H cross sections from ctsnHMQC spectra obtained from the same raw data as Figure 3 b. Traces on the left are processed without DBU, whereas traces on the right are processed using DBU. Traces (a) were taken from the corresponding 2 D spectra at 61.4 ppm, trace (b) at 71.5 ppm, and trace (c) at 92.2 ppm as indicated in Figure 3 b.

Taking advantage of the improved sensitivity a heteronuclear long‐range correlation experiment (HMBC)^21^, ^22^ was implemented in a spin‐noise‐detected fashion, ctsnHMBC. In Figure 5 the results of the first implementation of a ctsnHMBC experiment are compared with a standard pulsed HMBC experiment. While demonstrating a new breakthrough in spin‐noise‐detected NMR, the experiment clearly shows up its current limits, as only the two correlations, for which the chosen mixing delays match best can be unequivocally identified. The H4–C3 correlation, for example, is not detected due to insufficient signal‐to‐noise ratio.

**Figure 5:**
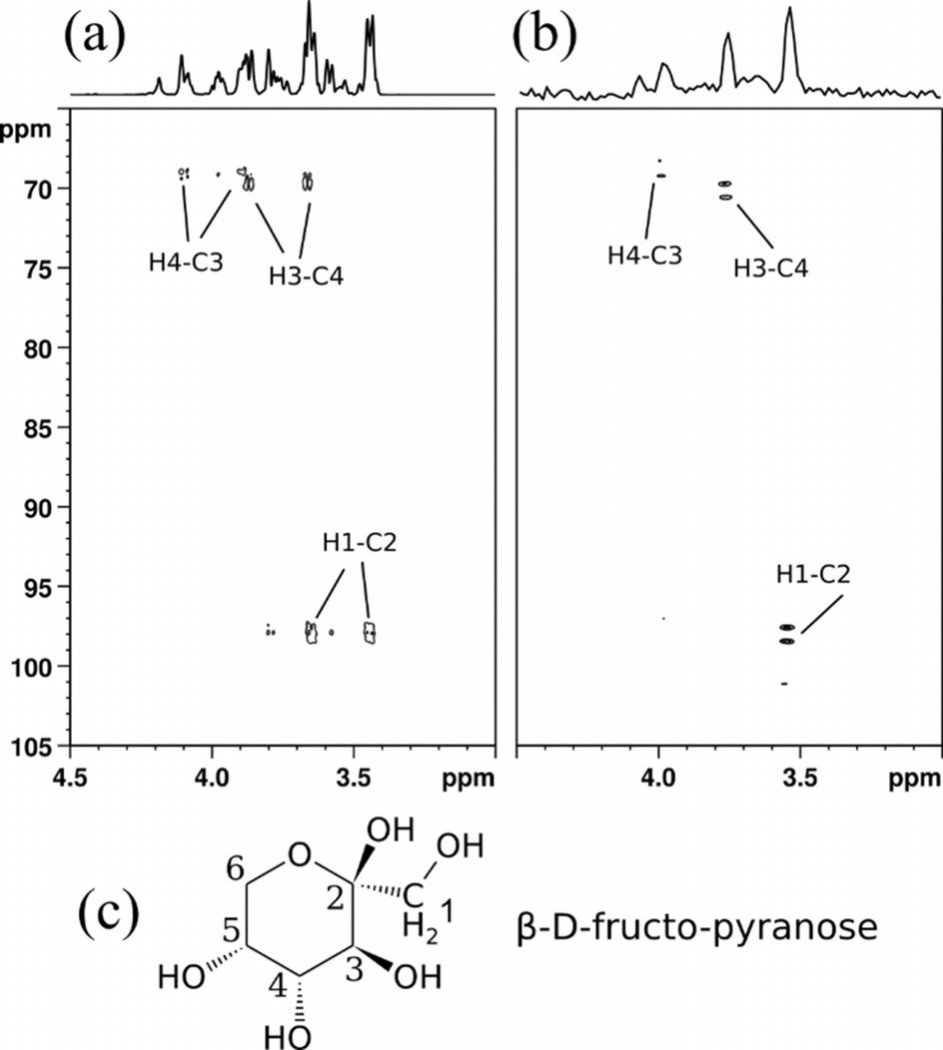
Pulsed and noise‐detected HMBC experiments of d‐fructose. The spectrum in panel (a) was obtained using a standard pulsed HMBC experiment (pulse program: ′hmbcgpndqf′)^23^ and is compared to the results of the ctsnHMBC experiment in panel (b). The recording time of the ctsnHMBC was 80 h; resolution(F1)=22.0 Hz; resolution(F2)=19.1 Hz. Panel (c) shows the numbering of the most abundant fructose isomer in aqueous solution.

Overall, the concept of two‐dimensional spin noise NMR spectroscopy could be extended significantly. The experimental schemes of spin‐noise‐detected 2 D NMR were substantially improved and adapted to avoid an artifact present on some spectrometer hardware, which is related to the active switching between a transmit and a receive state in the preamplifier electronics. This insight appears to be important, because this interference may influence the outcome of spin noise experiments drastically. Notably, it may increase the amount of uncorrelated noise background in the experiments relying upon cross‐correlation to distinguish between completely random and coherently transferred spin noise components.

We have shown that standard protocols of pulsed NMR, like phase cycling, pulsed field gradients and States or TPPI schemes can be used to improve the signal‐to‐noise ratio, achieve quadrature detection in the indirect dimension, and remove artifacts. These results demonstrate that many “tools of the trade” of traditional NMR can be applied for spin‐noise‐detected NMR spectroscopy.

The DBU fast acquisition scheme introduced here represents a paramount and unique acceleration of spin noise spectroscopy, because it achieves a tremendous speedup of the experiments, which is not possible in pulsed excitation NMR due to the dependence on longitudinal relaxation. Comparing the original experiment^1^ at equal recording times and on identical hardware, we have been able to achieve an increase by nearly a factor of three in signal‐to‐noise ratio by combining all improvements described here, which boils down to nearly one order of magnitude in experimental time. While the real benefit of this will only be of practical relevance, once a future “nano‐NMR spectrometer” is constructed, stochastic NMR excitation techniques using an RF noise source may also take advantage from the DBU principle.^24^ The DBU acceleration principle for coherent spectroscopic techniques is not limited to Faraday detection as in the application outlined here, it is likewise suitable to be incorporated in optical detection methods, as for example in the novel nano‐scale NMR detected indirectly by NV‐centers in diamonds.^25^

## Experimental Section

The substances used for demonstrating the spin noise NMR experiments were uniformly 99 at.% ^13^C enriched d‐glucose (Aldrich) for all HMQC‐type experiments and 99 at.% ^13^C enriched d‐fructose (Aldrich) for all HMBC‐type experiments. The solvent was D_2_O (Euriso‐Top, 99.90 at.% D). The concentration was 0.65 mol L^−1^ for the d‐glucose (as in the original experiment^1^) and 3.20 mol L^−1^ for d‐fructose. We note that spin‐noise‐detected experiments involving coherence transfer are best conducted in the regime of positive spin noise signal^8^, ^26^ to mitigate quenching by radiation damping. The validity of this condition was tested before the 2 D experiments by recording one dimensional spin noise spectra.

The effectiveness of the cross‐correlation processing is critically affected by transverse relaxation between the respective noise blocks, which was taken care of through optimization of the delay times in the pulse sequences as given below. This optimization was effected by prepending a single 1 μs ^1^H pulse to a train of 32 repetitions of the respective pulse sequence and maximizing cross peak intensities in the spectra processed identically to the spin noise spectra. The apparent fast transverse relaxation rates under the conditions given (e.g. T_2_^*^≅10 s^−1^ for the anomeric protons) are mostly owed to radiation damping, which can be suppressed during the coupling precession intervals *τ*_1_ by application of sign alternating pulsed field gradients.

Standard (Wilmad 535) 5 mm NMR sample tubes were used. Spectra were recorded at 313 K on a 700 MHz Bruker Avance III spectrometer with a cryogenically cooled TCI triple resonance (H,C,N,D) probe manufactured in 2011. Common parameters for both spectra in Figure 3 were duration of acquisition *t*_0_=*t*_2_=26.2 ms corresponding to a resolution of 19.1 Hz in F2; *t*_1_ increments were adjusted for a spectral width of 7044.5 Hz (40 ppm) in F1 and the maximum duration of *t_1_* was set to 22.7 ms corresponding to a resolution of 22.0 Hz in F1; *τ_1_*=3.45 ms and *τ*=22.7 ms. For the ctsnHMBC experiment of Figure 5 c the pulse sequence of Figure 2 d with *τ*_1_=55.6 ms; *τ*=22.7 ms and shaped (squared sine bell) pulsed field gradients with 2 % of the full amplitude (50 Tm^−1^) and 5 ms duration were used.

To mitigate long‐term instability (temperature, humidity, vibrations, etc.) of the spectrometer environment in general, all the experiments were recorded in multiple sections, which were combined during processing. Acquisition of each section lasted approx. 2 h and in between the recording of individual sections magnetic field corrections (Bruker's “topshim” procedure) were re‐optimized. For example, for an experiment with a total duration of 20 h, ten full 2 D experiments were recorded each lasting for about 2 h and finally combined. The increase in total recording time due to periodic automated shimming is less than 5 %. The script for the automatic acquisition and shimming sequence is shown in the Supporting Information.

## Conflict of interest

*The authors declare no conflict of interest*.

## Supporting information

As a service to our authors and readers, this journal provides supporting information supplied by the authors. Such materials are peer reviewed and may be re‐organized for online delivery, but are not copy‐edited or typeset. Technical support issues arising from supporting information (other than missing files) should be addressed to the authors.

Supplementary

## References

[1] K. Chandra , J. Schlagnitweit , C. Wohlschlager , A. Jerschow , N. Müller , J. Phys. Chem. Lett. 2013, 4, 3853–3856. 24294412 10.1021/jz402100g PMC3843499

[2] F. Bloch , Phys. Rev. 1946, 70, 460–474.

[3] T. Sleator , E. L. Hahn , C. Hilbert , J. Clarke , Phys. Rev. Lett. 1985, 55, 1742–1745. 10031911 10.1103/PhysRevLett.55.1742

[4] M. A. McCoy , R. R. Ernst , Chem. Phys. Lett. 1989, 159, 587–593.

[5] H. Kovacs , D. Moskau , M. Spraul , Prog. Nucl. Magn. Reson. Spectrosc. 2005, 46, 131–155.

[6] M. Guéron , J. L. Leroy , J. Magn. Reson. 1989, 85, 209–215.

[7] N. Müller , A. Jerschow , Proc. Natl. Acad. Sci. USA 2006, 103, 6790–6792. 16636281 10.1073/pnas.0601743103 PMC1458973

[8] M. T. Pöschko , V. V. Rodin , J. Schlagnitweit , N. Müller , H. Desvaux , Nat. Commun. 2017, 8, 13914. 28067218 10.1038/ncomms13914 PMC5227550

[9] J. Schlagnitweit , N. Müller , J. Magn. Reson. 2012, 224, 78–81. 23041799 10.1016/j.jmr.2012.09.002 PMC3611093

[10] M. Bechmann , N. Müller , Annu. Rep. NMR Spectrosc. 2017, 92, 199–226.

[11] M. T. Pöschko , B. Vuichoud , J. Milani , A. Bornet , M. Bechmann , G. Bodenhausen , S. Jannin , N. Müller , ChemPhysChem 2015, 16, 3859–3864. 26477605 10.1002/cphc.201500805 PMC4691331

[12] M. Nausner , J. Schlagnitweit , V. Smrečki , X. Yang , A. Jerschow , N. Müller , J. Magn. Reson. 2009, 198, 73–79. 19232505 10.1016/j.jmr.2009.01.019

[13] N. Müller , A. Jerschow , J. Schlagnitweit , eMagRes 2013, 2, 237–243..

[14] C. L. Degen , M. Poggio , H. J. Mamin , C. T. Rettner , D. Rugar , Proc. Natl. Acad. Sci. USA 2009, 106, 1313–1317. 19139397 10.1073/pnas.0812068106 PMC2628306

[15] W. P. Aue , E. Bartholdi , R. R. Ernst , J. Chem. Phys. 1976, 64, 2229–2246.

[16] A. Einstein , Arch. Sci. Phys. Nat. 1914, 37, 254–256;

[17] D. J. States , R. A. Haberkorn , D. J. Ruben , J. Magn. Reson. 1982, 48, 286–292.

[18] D. Marion , K. Wüthrich , Biochem. Biophys. Res. Commun. 1983, 113, 967–974. 6307308 10.1016/0006-291x(83)91093-8

[19] V. Sklenar , J. Magn. Reson. 1995, 114, 132–135.

[20] J. Schlagnitweit , M. Hornicakova , G. Zuckerstätter , N. Müller , ChemPhysChem 2012, 13, 342–346. 22095747 10.1002/cphc.201100525 PMC3298640

[21] W. Schöfberger , J. Schlagnitweit , N. Müller , Annu. Rep. NMR Spectrosc. 2011, 72, 1–60.

[22] A. Bax , M. F. Summers , J. Am. Chem. Soc. 1986, 108, 2093–2094.

[23] TopSpin v3.5pl5, Bruker-Biospin, Rheinstetten, Germany **2016**.

[24] B. Blümich , J. Magn. Reson. 1990, 90, 535–543.

[25] N. Aslam , M. Pfender , P. Neumann , R. Reuter , A. Zappe , F. Fávaro de Oliveira , A. Denisenko , H. Sumiya , S. Onoda , J. Isoya , J. Wrachtrup , Science 2017, 357, 67–71. 28572453 10.1126/science.aam8697

[26] P. Giraudeau , N. Müller , A. Jerschow , L. Frydman , Chem. Phys. Lett. 2010, 489, 107–112.

