# Supplementary material for: Spin‐Noise‐Detected Two‐Dimensional Nuclear Magnetic Resonance at Triple Sensitivity
Source: Chemphyschem. 2018 Feb 20;19(8):907–12. doi: 10.1002/cphc.201800008 (PMC5915744; doi:10.1002/cphc.201800008)
Supplement: Supplementary file 1 — Supplementary [file CPHC-19-907-s001.pdf]

CHEMPHYSCHEM

Supporting Information

Spin-Noise-Detected Two-Dimensional Nuclear Magnetic Resonance at Triple Sensitivity

Stephan J. Ginthör,[a] Kousik Chandra,[a, b] Matthias Bechmann,[a] Victor V. Rodin,[a] and Norbert Müller*[a, c]

cphc_201800008_sm_miscellaneous_information.pdf

Author Contributions

S.G. Conceptualization: Supporting; Data curation: Equal; Formal analysis: Equal; Investigation: Lead; Methodology: Supporting; Software: Lead; Validation: Lead; Visualization: Lead; Writing – original draft: Supporting; Writing – review & editing: Equal

K.C. Data curation: Supporting; Funding acquisition: Equal; Investigation: Equal; Methodology: Supporting; Project administration: Equal; Writing – original draft: Supporting

M.B. Data curation: Supporting; Formal analysis: Supporting; Investigation: Supporting; Methodology: Equal; Software: Supporting; Validation: Supporting; Visualization: Supporting; Writing – original draft: Supporting

V.R. Data curation: Supporting; Investigation: Supporting; Methodology: Supporting; Validation: Supporting; Visualization: Supporting

N.M. Conceptualization: Lead; Data curation: Supporting; Formal analysis: Equal; Funding acquisition: Lead; Investigation: Equal; Methodology: Lead; Project administration: Lead; Resources: Lead; Software: Supporting; Supervision: Lead; Validation: Supporting; Visualization: Supporting; Writing – original draft: Lead; Writing – review & editing: Lead.

Table of Contents

Experimental Procedures ...2
Assessment of Switching Artifacts ...5
Spin Noise vs. Circuit Noise ...6
References ...6

Experimental Procedures

As mentioned in the main text, we modified the implementation of spin noise acquisition of previous publications[1–3] to avoid a hardware dependent artifact, which had been absent in our previous experiments. The artifact is generated, if one-dimensional spin noise spectra are recorded, noise block by noise block, while activating and deactivating the receiver channel for each block. The way one-dimensional spin noise spectra are processed is as follows: All the noise blocks are Fourier transformed individually to give power spectra and are co-added after that (see Figure S1).

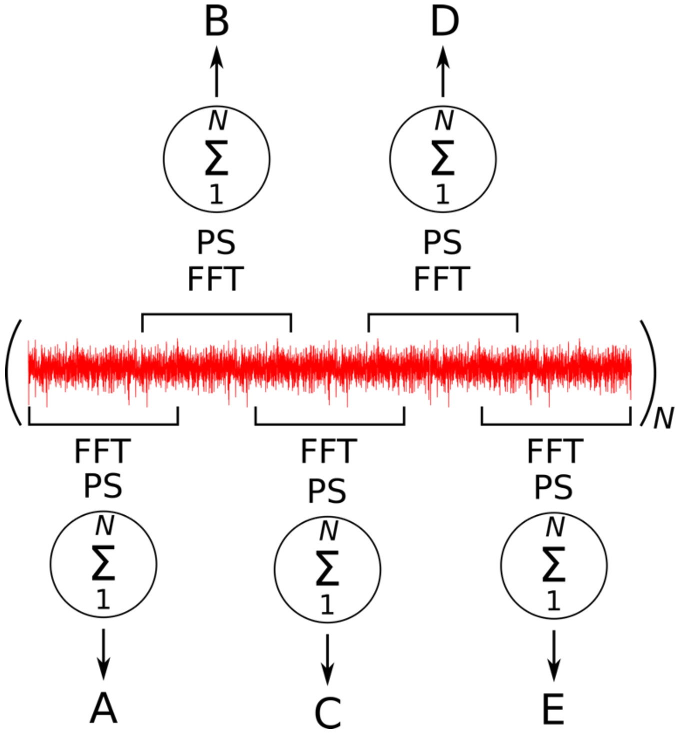

Figure S1. Scheme of one-dimensional spin noise acquisition, where *N* long noise blocks are recorded. Depending on the required resolution each noise block can either be Fourier transformed as a whole before addition of the N resulting power spectra or split into overlapping sub-blocks[8] before Fourier transformation, allowing one to adjust resolution according to the sample properties. In order to assess the time dependent influence of the transmit/receive switch, the corresponding sub-blocks (A to E) of each of the N acquired original blocks were processes and co-added separately. By comparing noise spectra obtained from the different sub-blocks the time-dependent components can be identified.

The artifact was first observed on newer hardware using a preamplifier with active transmit/receive switching as an apparent dependence of the spin noise line shape on the duration of the original noise blocks, which cannot be explained by a change in resolution or signal-to-noise, as shown in Figure S5. The spectra shown were obtained on a 500 MHz Bruker Avance III instrument with an ambient temperature probe and a low noise preamplifier. In addition, a continuous phase shifter (ARRA, DN2448A)[4] was placed between the probe and the preamplifier and adjusted to give a pure negative absorptive spin noise signal[5–7]. We note that this adjustment is not a requirement for the occurrence of the artifact. In Figure S5 one can observe that a positive contribution to the line shape exists for noise blocks acquired early after the start of the acquisition. Under these circumstances the spin noise signal is negative (i.e. less than thermal circuit noise), while the artifact contribution is always has positive power. This additional signal is not observed when adding the phase sensitive noise blocks, as it has random phase.
The pulse sequence programming for the modified acquisition scheme for ctsnHMQC is shown as Figure S2 and that of ctsnHMBC is shown in Figure S3. Figure S4 depicts the automation script used for intermediate shimming in long spin noise acquisitions.

```
;ctsnHMQC
;Authors: S. J. Ginthoer, M. Bechmann, K. Chandra, N. Mueller
;Date: 9.5.2016

;uses phase cycle
;uses States for indirect quad. detection, phase increments stored separately
;uses TPPI for displacement of unmodulated signal artifacts

;SET THIS MANUALLY:
;set cnst1 to the desired TD2 (FID/noise block length) value
;set TD2 to the maximal buffer size or (TD2 = cnst1 (TD2 for this experiment) * noise repetitions)
;adjust SW (dwell time) to maximize anavpt
;HOWTO:
;1) set SW to desired value
;2) set DIGMOD to digital to see how many points are decimated
;3) ajust SW so that the decimated points equal or are only slightly greater than a given power of two
;4) set this power of two value in the anavpt below

;uses continuous aquisition
;no preamp switching during experiment avoids T/R switch effect

#include <Avance.incl>
#include <De.incl>

dwellmode explicit                        ;set dwellmode to explicit
"anavpt = 4096"                           ;must be set manually to maximize points during dwell time
                                          ;you may need adjust SW (anavpt can only be a power of 2)

define loopcounter td2h
define loopcounter bufferCounter
define loopcounter td1h

"td2h = cnst1/2"                          ;for each acquisition 2 real points are recorded, so td2/2
"bufferCounter = td/cnst1"                ;initiate bufferCounter
"td1h = td1/2"                            ; reset td1 to real td1 / bufferCounter
"d12 = dw*2"                              ;this is the dwell time

"in0=inf1/2"                              ;in/decrement for t1
"in10=in0"

"p4=2*p3"                                 ;90 and 180 degree pulses for 13C

"d26=1s/(cnst2*4)"                        ; JCH
"d10=1s/(cnst4)"                          ; JCC 50..60
"d0=2u"
"d30=3m"                                  ;delay to initiate disk write
"d31=20ms"                                ;delay to quench transient effect


        ze
2       d30     pl12:f2
        ACQ_START1(ph30,ph31)             ;aquisition is started
        1u      REC_UNBLK                 ;unblank receiver path (T/R switch to T mode)
3       d31                               ;wait for d31 let artifact decay
4       0.1u    cpds2:f2                  ;enable decoupling on 13C
5       d12     DWL_CLK_ON
        0.1u    DWL_CLK_OFF
        lo to 5 times td2h                ;this loop records td2h*2 datapoints (td2h complex datapoints)
        d26     do:f2                     ;this begins the mixing period, disable decoupling
        d26     pl2:f2
        (p3 ph2):f2
        d0
        (p4 ph2):f2
        d10
        (p3 ph3):f2
        d26     pl12:f2                   ;this ends the mixing period
        d26

        lo to 4 times bufferCounter       ;loop for the noise block repetitions
        1u      REC_BLK
        100u    eoscnp1
        500m    wr #0 if #0 zd ip3        ;write buffer out, huge FID so long delay, increment phase and delays
        lo to 2 times 2                   ;loop twice for States TPPI
        d30     id0
        d30     dd10
        lo to 2 times td1h                ;loop for the F1 increments

exit

;receiver phases NB: phase steps are stored separately.
ph30=0
ph31=0

;pulse phases
ph1=0
ph2=0
ph3=0

;anavpt                                     points decimated during dw*2 (aquisition of two datapoints)
;td2h                                        half of TD2 corresponding to number of acquired complex points
;cnst1                                       used to set the real length of one FID (usually TD2)
;noise_repetitions                           number of noise blocks recorded for each phase and t1 increment
;td                                          this is the TD2 parameter from the foreground settings (multiple noise
                                            ;blocks are stored in one FID for this experiment)
;td1h                                       half of the points of TD1 (because of States TPPI)
;d12                                         delay for DWL_CLK_ON/OFF (dw*2)
;inf1                                        the delay calculated for t1 increment
;in0                                         increment of d0, in0=inf1/2, this is because of experimental properties,
                                            ;the evolution happens twice as fast so this delay is halfed to correct
                                            ;ppm scale
;in10                                        same as in0, but for d10 instead of d0, used as decrement
;p3                                          length of 90 degree 13C pulse
;p4                                          length of 180 degree 13C pulse
;ph2                                         phase of the 13C pulses
;ph3                                         phase of the last 90 degree 13C pulse, subjected to phase cycling for
                                            ;States TPPI
;ph30                                        phase of the receiver
;ph31                                        phase of the mixing frequency/transmitter frequency
;d26                                         delay for heteronuclear J-coupling constant
;cnst2                                       heteronuclear J-coupling constant
;d0,d10                                      constant time delays for homonuclear JCC coupling
;cnst4                                       homonuclear JCC coupling constant
;d30                                         hardware delay
;d31                                         delay to relax the transient effect
;pl12                                        decoupling power level
;pl2                                         power level of the 13C pulses
```

Figure S2. Pulse program **(tested with TopsSpin 3.5pl5)** of a ctsnHMQC experiment avoiding T/R switching artifacts.

```
;ctsnHMBC
;Authors: S. J. Ginthoer, M. Bechmann, K. Chandra, N. Mueller
;Date: 2.10.2016

;uses gradients + phase cycle
;uses States for indirect quad. detection, phase increments stored separately

;SET THESE MANUALLY:
;set cnst1 to the desired TD2 (FID/noise block length) value
;set TD2 to the maximal buffer size or (TD2 = cnst1 (TD2 for this experiment) * noise repetitions)
;adjust SW (dwelltime) to maximize anavpt
;HOWTO:
;1) set SW to desired value
;2) set DIGMOD to digital to see how many points are decimated
;3) ajust SW so that the decimated points equal or are only slightly greater than a given power of two
;4) set this power of two value in the anavpt below

;uses continuous aquisition
;no preamp switching during experiment avoids T/R switch effect

#include <Avance.incl>
#include <De.incl>

dwellmode explicit                        ;set dwellmode to explicit

"anavpt = 4096"                           ;must be set manually to maximize points during dwell time
                                          ;you may need adjust SW (anavpt can only be a power of 2)

define loopcounter td2h
define loopcounter bufferCounter
define loopcounter td1h

"td2h = cnst1/2"                          ;for each acquisition 2 real points are recorded, so td2/2
"bufferCounter = td/cnst1"                ;initiate bufferCounter
"td1h = td1/4"                            ; reset td1 to real td1 / bufferCounter
"d12 = dw*2"                              ;this is the dwell time

"in0=inf1/2"                              ;in/decrement for t1
"in10=in0"

"p4=2*p3" ;90 and 180 degree pulses for 13C

"d26=1s/(cnst2*4)"                        ; JCH
"d10=1s/(cnst4)"                          ; JCC 50..60
"d0=2u"
"d30=3m"                                  ;delay to initiate disk write
"d31=20ms"                                ;delay to quench transient effect
"d27=d26-p16-d16-3u"

        ze
2       d30     pl12:f2
        ACQ_START1(ph30,ph31)              ;aquisition is started
        1u      REC_UNBLK                  ;unblank receiver path (T/R switch to T mode)
3       d31                                ;wait for d31 let artifact decay
4       0.1u    cpds2:f2                   ;enable decoupling on 13C
5       d12     DWL_CLK_ON
        0.1u    DWL_CLK_OFF
        lo to 5 times td2h                 ;this loop records td2h*2 datapoints (td2h complex datapoints)
        3u      do:f2                      ;this begins the mixing period, disable decoupling
        p16:gp1
        d16
        d27
        d26     pl2:f2
        (p3 ph4):f2
        d0
        (p4 ph2):f2
        d10
        (p3 ph3):f2
        d26     pl12:f2                    ;this ends the mixing period
        d27
        3u
        p16:gp1*-1
        d16

        lo to 4 times bufferCounter        ;loop for the noise block repetitions
        1u      REC_BLK
        100u    eoscnp1
        500m    wr #0 if #0 zd ip3         ;write buffer out, huge FID so long delay, increment phase and
                                           ;delays
        lo to 2 times 2                    ;loop twice for States TPPI
        d30     rp3
        d30     ip4*2
        lo to 2 times 2
        d30     id0
        d30     dd10
        lo to 2 times td1h                 ;loop for the F1 increments

exit

;receiver phases NB: phase steps are stored separately.
ph30=0
ph31=0

;pulse phases
ph1=0
ph2=0
ph3=0
ph4=0

;anavpt                                     points decimated during dw*2 (aquisition of two datapoints)
;td2h                                       half of TD2 corresponding to number of acquired complex points
;cnst1                                      used to set the real length of one FID (usually TD2)
;noise_repetitions                          number of noise blocks recorded for each phase and t1 increment
;td                                         this is the TD2 parameter from the foreground settings (multiple noise
                                            ;blocks are stored in one FID for this experiment)
;td1h                                       half of the points of TD1 (because of States TPPI)
;d12                                        delay for DWL_CLK_ON/OFF (dw*2)
;inf1                                       the delay calculated for t1 increment
;in0                                        increment of d0, in0=inf1/2, this is because of experimental properties,
                                            ;the evolution happens twice as fast so this delay is halfed to correct
                                            ;ppm scale
;in10                                       same as in0, but for d10 instead of d0, used as decrement
;p3                                         length of 90 degree 13C pulse
;p4                                         length of 180 degree 13C pulse
;ph2                                        phase of the 13C pulses
;ph3                                        phase of the last 90 degree 13C pulse, subjected to phase cycling for
                                            ;States TPPI
;ph30                                       phase of the receiver
;ph31                                       phase of the mixing frequency/transmitter frequency
;d26                                        delay for heteronuclear J-coupling constant
;cnst2                                      heteronuclear J-coupling constant
;d0,d10                                     constant time delays for homonuclear JCC coupling
;cnst4                                      homonuclear JCC coupling constant
;d30                                        hardware delay
;d31                                        delay to relax the transient effect
;pl12                                       decoupling power level
;pl2                                        power level of the 13C pulses
```

Figure S3. Pulse program **(tested with TopsSpin 3.5pl5)** of a ctsnHMBC experiment avoiding T/R switching artifacts.

```
int start = 2, end = 10, i = 0;

for(i=start;i<=end;i++){
  WRA(i);
  expno = i;
  SETCURDATA
  ZG
  CPR_exec("topshim tunea", WAIT_TERM);
}

QUIT
```

Figure S4. AU script (tested with TopSpin 3.5pl5) for recording multiple experiments with shimming in between.

Assessment of Switching Artifacts

As can be seen in Figure S5, the time-dependent component is strongest for sub-blocks taken from the beginning of each noise block and it decays within 7–20 ms (probe and sample dependent). This effect has been traced back to the activation of the transmit/receive (T/R) switch in the preamplifier.[5] The impedance change caused by the T/R switching apparently acts like a minute random pulse excitation. The strength and persistence of this effect is highly dependent on the probe and the type of preamplifier. It was found to be independent of the carrier frequency.

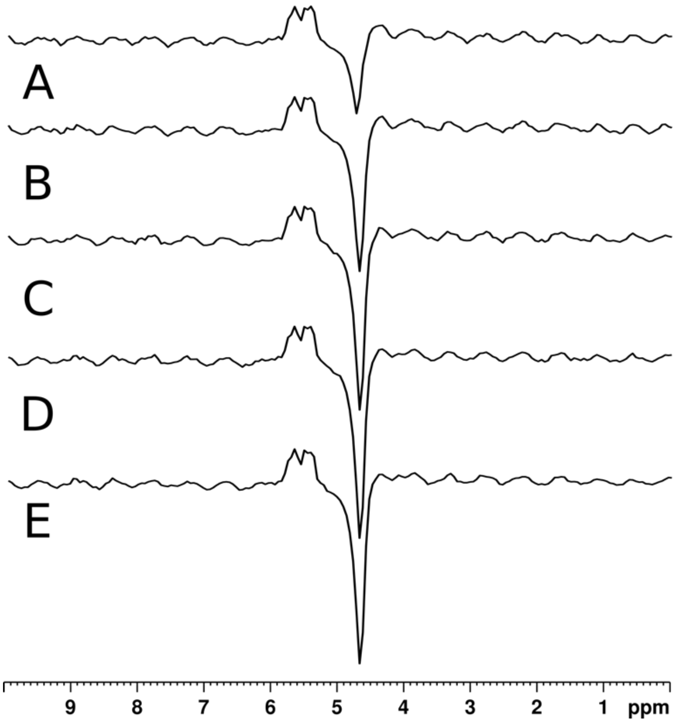

Figure S5. Five 500 MHz ^{1}H spin noise spectra of a sample of 90% H_2O / 10% D_2O. The sub-blocks were extracted from the original noise blocks as indicated in Fig. S1 and processed accordingly. The original noise blocks consisted of 4096 data points, which were recorded over a period of 80 ms and then divided into 5 equally spaced overlapping blocks of 1024 data points (corresponding to 20 ms) as visualized in Fig. S1. Spectra A to E were obtained from the equidistant 1024 data point contiguous sub-blocks corresponding to delay times of 0 ms to 64 ms after the start of the original block. The proton spin noise signal of H_2O is the negative peak at 4.7 ppm. The positive signals centered at 5.5 ppm are artifacts originating outside the spectrometer, most likely from a nearby TV station. The carrier frequency was set to 3.5 ppm.

For one-dimensional experiments this can be trivially accomplished by extending each noise block to the hardware maximum and discarding the noise recorded within 5×T_2^* from the start of acquisition. For two-dimensional spin-noise-detected NMR spectra, where the acquisition of multiple non-contiguous individual noise blocks is required, the pulse programs have to be rewritten to prevent engaging the T/R switch. In Figure S6 we show a pulse program for a ctsnHMBC experiment, programmed such that the T/R switch is only activated once at the beginning of each burst of DBU-acquisition blocks. After the T/R switch is engaged, there is an additional delay of 20 ms, to allow for the artifact to decay. In order to avoid this potential source of interference in spin-noise-detected experiments, the programming of spin noise acquisition schemes was modified. Instead of activating the T/R switch for each noise block (Figure S6, upper panel), the switch remains in the receive state continuously (in practice, until the hardware buffer is full, Figure S6, lower panel).

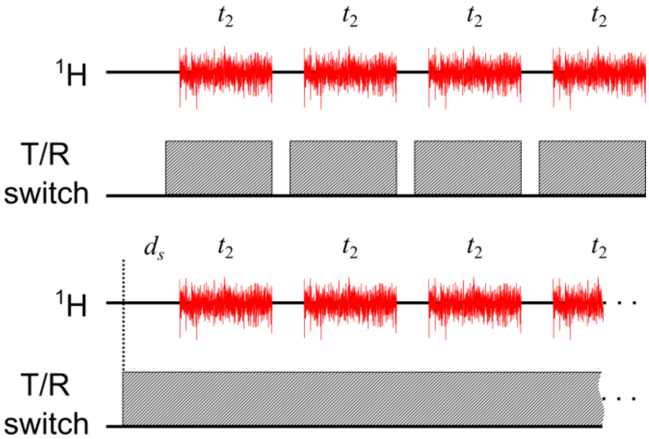

Figure S6. The upper scheme shows the approach to transmit/receiver switch control used in previous experiments. Before each FID or noise block the switch is opened and after the acquisition the switch is closed again. The engagement of the switch however may cause a small pulse-like event at the beginning of the noise block, which shows up as an always positive distortion of the spin noise line shape, as shown in Fig. S2. This effect is only noticeable in spin noise experiments, due to the low amplitude of the phenomenon. Therefore, multiple FIDs or noise blocks are acquired with the switch continuously in the receive state, as shown in the lower scheme. This circumvents the problem if a delay d_s of duration 5T_2^*) is inserted after the T/R switch is activated.

On the spectrometer systems we used here (Bruker Avance III) with the software version used (Topspin 3.5pl5), implementing acquisition as shown in the pulse program of Figure S6 requires one to set the "acquisition mode" to "analog" (i.e. disable the real time digital signal processor) to avoid digital filtering artifacts to occur when multiple noise blocks are concatenated. As a consequence of this "analogue" acquisition mode, the acquired signals contain a direct current offset, that may drift over the course of an experiment (20 h), and which may lead to a visible center artifact in the final spectrum. To mitigate this interference, the individual noise blocks are linearly offset corrected, i.e. their average is subtracted from each data point. There is one caveat with this approach: any genuine NMR signal at the center will be removed as well. The data presented here in this Supplementary Information were recorded on a Bruker 500 MHz Avance III spectrometer, equipped with a BBI probe. On our Bruker 700 MHz Avance III spectrometer with a TCI triple resonance cryoprobe, the effect is weaker and decays faster, but still might be problematic for spin noise experiments. In spin-noise-detected experiments using repetitive T/R switching with the acquisition and cross-correlation processing scheme outlined in the main text or applying the original acquisition scheme[1] the effect of the T/R switching artifact with is an increase of the uncorrelated noise as compared to the correlated spin noise signal, owed to the fact the T/R switch artifacts are not correlated between subsequently recorded noise blocks.

Spin Noise vs. Circuit Noise

While we have used the terms "pure spin noise" and "absorbed circuit noise" in previous work,[9] the distinction between spin noise and circuit noise may be misleading and is not as clear-cut as it might seem, in particular on high-Q probes. Due to the strong coupling between the RF-circuit and the spins, one observes a collective phenomenon. The major source of noise not originating from the nuclear spins in our setup is the preamplifier noise, which has been extensively covered in our recent paper.[10] As explained there, preamplifier noise causes line shape changes in the spin noise signals and, most importantly, quantitatively explains the frequency shifts in the tuning curves observed previously.[11-16] We assured that there was no additional source of noise in the experimental setup used here, as the spin noise response is not affected by changing the cabling between the cold (77 K) pre-amplifier and the "warm" (ambient temperature) pre-amplifier including replacement of the RF-pulse cable by 50 Ohm terminators.

References

[1] K. Chandra, J. Schlagnitweit, C. Wohlschlager, A. Jerschow, N. Müller, *J. Phys. Chem. Lett.* **2013**, *4*, 3853–3856.
[2] N. Müller, A. Jerschow, J. Schlagnitweit, *eMagRes* **2013**, *2*, 237–243.
[3] N. Müller, A. Jerschow, *Proc. Natl. Acad. Sci.* **2006**, *103*, 6790–6792.

[4] Purchased from “ARRA, Inc.” see http://arra.com/phase_shifters.html**, 2016.**

[5] G. Ferrand, G. Huber, M. Luong, H. Desvaux, *J. Chem. Phys.* **2015**, *143*, 094201.

[6] M. Nausner, J. Schlagnitweit, V. Smrečki, X. Yang, A. Jerschow, N. Müller, *J. Magn. Reson.* **2009**, *198*, 73–79.

[7] M. T. Pöschko, V. V. Rodin, J. Schlagnitweit, N. Müller, H. Desvaux, *Nat. Commun.* **2017**, *8*, 13914.

[8] H. Desvaux, D. J. Y. Marion, G. Huber, P. Berthault, *Angew. Chem.* **2009**, *121*, 4405--4407; *Angew. Chem. Int. Ed.* **2009**, *48*, 4341–4343.

[9] P. Giraudeau, N. Müller, A. Jerschow, L. Frydman, *Chem. Phys. Lett.* **2010**, *489*, 107-112

[10] M. T. Pöschko, V. V. Rodin, J. Schlagnitweit, N. Müller, H. Desvaux, *Nat. Commun.* **2017**, *8*, 13914.

[11] D. J-Y. Marion, H. Desvaux, *J. Magn. Reson.* **2008**, *193,* 153-157.

[12] M. Nausner, J. Schlagnitweit, V. Smrečki, X. Yang, A. Jerschow, N. Müller, *J. Magn. Reson.* **2009**, *198*, 73–79.

[13] E. Bendet-Taicher, N. Müller, A. Jerschow, *Conc. Magnet. Reson. B* **2014**, *44B*, 1–11.

[14] M. T. Pöschko, J. Schlagnitweit, G. Huber, M. Nausner, M. Horničáková, H. Desvaux, N. Müller, *ChemPhysChem* **2014,** *15*, 3639–3645.

[15] G. Ferrand, M. Luong, G. Huber, H. Desvaux, *Conc. Magnet. Reson. B* **2015**, *45B*, 59–68.

[16] M. T. Pöschko, B. Vuichoud, J. Milani, A. Bornet, M. Bechmann, G. Bodenhausen, S. Jannin, N. Müller, *ChemPhysChem* **2015**, *16*, 3859–3864.

Author Contributions

Experiments were performed with equal contributions by S.G., C.K., and V.V.R.; S.G. and M.B. handled the programming and data analysis, the manuscript was written by S.G., M.B. and N.M., N.M. and C.K. acquired the funding.
